# Human placental extract, melsmon, suppresses stress-induced neuroinflammation and peripheral inflammation in a mouse model of depression

**DOI:** 10.3389/fphar.2026.1786578

**Published:** 2026-06-01

**Authors:** Naomi Oka, Kazuya Shimada, Azusa Ishii, Yohei Watanabe, Kazuhiro Kondo, Takeshi Yamamoto

**Affiliations:** 1 Department of Virology, The Jikei University School of Medicine, Tokyo, Japan; 2 Department of Fatigue Science, The Jikei University School of Medicine, Tokyo, Japan; 3 Corporate Planning Division, Melsmon Pharmaceutical Co. Ltd., Tokyo, Japan

**Keywords:** depression, inflammation, melsmon, neuroinflammation, placental extract, stress

## Abstract

Neuroinflammation and hyperactivation of the hypothalamic–pituitary–adrenal (HPA) axis triggered by stress have been implicated in the pathophysiology of depression, highlighting the need for novel therapeutic approaches targeting these mechanisms. Placental extracts have been reported in clinical studies to alleviate fatigue and depressive symptoms, suggesting their potential to modulate stress-induced neuroinflammatory responses and HPA-axis activation, although this has not been adequately investigated. In this study, we used a stress-sensitive depression model mouse (SITH-1 mouse), which expresses the human herpesvirus 6 (HHV-6)-derived protein SITH-1 specifically in astrocytes, and subjected it to mild stress via water immersion cage stress (WICS) to induce inflammatory cytokine expression in the brain and peripheral organs. We further examined the effects of intramuscular administration of the human placental extract “Melsmon” (20 μL per injection, administered every other day for four doses) on neuroinflammation and HPA-axis hyperactivity in this model. As a result, WICS exposure led to increased expression of inflammatory cytokines in the brain and in peripheral organs such as the liver, colon, and heart, along with elevated expression of steroidogenic acute regulatory protein (StAR), a marker of HPA-axis activation, in the adrenal gland. These changes were suppressed by Melsmon treatment. Collectively, our findings suggest that Melsmon exerts anti-inflammatory effects in both central and peripheral tissues, potentially contributing to the stabilization of the HPA axis and attenuation of stress-related inflammatory responses involved in the pathogenesis of depression. These findings suggest that placental extracts may serve as a novel therapeutic strategy for treating stress-related neuroinflammatory disorders such as depression.

## 1 Introduction

Depression is a psychiatric disorder that arises from a complex interplay of multiple factors, including chronic stress, genetic predisposition, environmental influences, and neurophysiological changes (Tafet and Nemeroff, 2016). Although the pathophysiology of depression remains incompletely understood, the monoamine hypothesis has long been dominant. However, recent studies have proposed more multifactorial hypotheses, including hyperactivation of the hypothalamic–pituitary–adrenal (HPA) axis, neuroinflammation, reduced neurogenesis, and impaired neuroplasticity (Dean and Keshavan, 2017; Ferrari and Villa, 2017; Cui et al., 2024), with particular attention being paid to the “neuroinflammation hypothesis” and the “neuroplasticity hypothesis”.

Neuroinflammation refers to the activation of glial cells such as microglia and astrocytes, leading to the production and release of inflammatory cytokines (e.g., IL-1β, IL-6, TNF-α) in the brain (Kwon and Koh, 2020). In animal models, chronic stress has been shown to activate microglia via the innate immune receptors TLR2/4, resulting in increased production of inflammatory cytokines that impair neuronal plasticity and induce depression-like behavior (Nie et al., 2018). Additionally, stress-induced activation of the sympathetic nervous system promotes the secretion of catecholamines and activates TLR4, thereby enhancing NF-κB signaling and upregulating the transcription of pro-inflammatory cytokines (Johnson et al., 2005; Gárate et al., 2013; Kuebler et al., 2015). In parallel, stress robustly activates the hypothalamic–pituitary–adrenal (HPA) axis, leading to sustained glucocorticoid release. Although glucocorticoids exert anti-inflammatory effects under physiological conditions, prolonged HPA-axis activation can lead to glucocorticoid resistance in immune cells, thereby impairing negative feedback regulation and promoting inflammatory cytokine production (Walsh, Bovbjerg, and Marsland, 2021; Hassamal, 2023).

These findings suggest that stress-induced neuroinflammatory processes, particularly when sustained, may contribute to the pathogenesis of depression (Koo and Wohleb, 2021). Inflammatory cytokines have been shown to suppress brain-derived neurotrophic factor (BDNF) expression and impair neurogenesis, dendritic growth, and synaptogenesis, thereby disrupting neural circuits involved in emotional regulation (Khairova et al., 2009; Calabrese et al., 2014; Zheng et al., 2021). Indeed, clinical studies have reported elevated levels of inflammatory cytokines in the blood of patients with depression (Maes et al., 1992; Zorrilla et al., 2001), suggesting that the stress-induced neuroinflammatory mechanisms observed in animal models may also be relevant to the pathophysiology of human depression. Based on this background, there is growing interest in developing antidepressants that target neuroinflammation and neuroplasticity. For example, glutamatergic agents such as ketamine and psilocybin have been reported to exert rapid antidepressant effects in treatment-resistant depression by promoting neuroplasticity (Rosas-Sánchez et al., 2024). Furthermore, anti-inflammatory compounds such as non-steroidal anti-inflammatory drugs (NSAIDs), cytokine inhibitors, and dipeptides that suppress microglial activation have demonstrated antidepressant-like effects (Kohler et al., 2016; Ano et al., 2019).

Our previous work demonstrated that the human herpesvirus 6 (HHV-6)-derived protein SITH-1, expressed during latent infection, activates the HPA axis and induces depression-like behavior in mice (Kobayashi et al., 2020). Mice expressing SITH-1 exhibit heightened stress sensitivity, showing depression-like behavior in response to mild stress. However, it remains unclear whether this model also displays neuroinflammatory responses, and the relationship between SITH-1 expression and neuroinflammation has not been fully explored. Given the established link between HPA-axis dysregulation and immune activation, we hypothesized that SITH-1 expression may create a stress-vulnerable state in which stress exposure triggers sustained inflammatory responses in the brain.

Among the emerging therapeutic bioproducts, placental extracts derived from mammalian placentae (e.g., human, horse, pig) have attracted increasing attention. These extracts, known to possess anti-inflammatory properties (Akagi et al., 2016; Ghoneum and El-Gerbed, 2021), have been used as pharmaceuticals and dietary supplements, particularly in several Asian countries (Yi Pan et al., 2017; Shen et al., 2022; Gwam et al., 2023). Clinical studies have suggested that various placental extracts may alleviate fatigue (Lee et al., 2012), improve osteoarthritis symptoms (Park and Cho, 2017), and reduce depressive symptoms (Kovalenko and Atalyan, 2016), indicating potential central nervous system activity of these extracts. One such extract, Melsmon®, a human placental preparation, has been reported to improve depressive symptoms and emotional instability in clinical trials (Kovalenko and Atalyan, 2016).

In this study, we investigated whether SITH-1–mediated stress vulnerability is accompanied by enhanced stress-induced inflammatory responses by applying water immersion cage stress (WICS) to SITH-1 mice and quantifying inflammatory cytokine expression in the brain and peripheral organs. We further evaluated whether administration of the human placental preparation Melsmon could attenuate these inflammatory responses. By focusing on the interplay between stress sensitivity, neuroinflammation, and HPA-axis activation, this study aimed to provide foundational evidence supporting the potential therapeutic application of placental preparations in stress-related psychiatric disorders.

## 2 Materials and methods

### 2.1 Animals

All experiments were conducted using male C57BL/6NCrSlc mice aged 8–9 weeks (body weight 22–27 g; n = 3‐5 per group), obtained from Sankyo Laboratories (Tokyo, Japan). Mice were housed under standard laboratory conditions with a 12-h light/dark cycle (lights on at 8:00 a.m.) and given *ad libitum* access to food and water. Mice were randomly assigned to experimental groups. All experimental procedures were approved by the Animal Experiment Committee of Jikei University School of Medicine and performed in accordance with institutional guidelines for animal care.

### 2.2 Production of recombinant adenovirus vectors

Recombinant adenovirus vectors were prepared as described previously (Kobayashi et al., 2020). An adenovirus expressing the HHV-6-derived SITH-1 protein under the control of the astrocyte-specific GFAP promoter was constructed using an adenovirus expression vector kit (Takara Bio Inc., Shiga, Japan) according to the manufacturer’s protocol. The GFAP promoter and PCR-amplified SITH-1 gene were cloned into an adenoviral cosmid vector (Ad-GFAP-SITH1) by standard molecular biology techniques. Recombinant adenoviruses (SITH-Ad or Vector-Ad) were generated by transfecting HEK293 A cells with the Ad-GFAP-SITH1 cosmid and a helper cosmid (pAxcwit) lacking the target gene. Viruses were purified using the Adeno-X Virus Purification Kit (Takara Bio Inc.), and viral titers were determined using the Adeno-X Rapid Titer Kit (Takara Bio Inc.).

### 2.3 Nasal inoculation of adenovirus vectors

Intranasal administration of adenovirus vectors was performed as previously described (Kobayashi et al., 2020). Briefly, male C57BL/6 mice aged 8–9 weeks were anesthetized with 3% isoflurane. Recombinant adenoviruses were diluted in sterile distilled water (not isotonic buffer). A total volume of 20 μL of viral solution (containing 1 × 10^9^ infectious units [ifu]/mL of either SITH-Ad or Vector-Ad) was applied dropwise to the nostrils in multiple aliquots. The solution was passively inhaled into the nasal cavity by spontaneous breathing.

### 2.4 Water immersion cage stress (WICS)

Water immersion cage stress (WICS) was applied as a prolonged stress paradigm. Mice were placed in standard cages containing 1 cm of water at the bottom without bedding and maintained under these conditions for 30 h (from 10:00 to 16:00 the following day). Animals had free access to food and water during the exposure period. WICS was initiated on day 6 after SITH-1 administration, as neurobiological alterations, including olfactory bulb apoptosis, reduced hippocampal neurogenesis, and HPA-axis activation, have been shown to occur within 7 days in this model (Kobayashi et al., 2020). After 30 h of water exposure, mice were returned to standard cages with dry bedding and allowed to recover for 18 h (from 16:00 to 10:00 the next day). Following the recovery period, mice were deeply anesthetized with an overdose of isoflurane and euthanized. Brain and peripheral organs were immediately collected for subsequent analyses. This stress paradigm was selected because preliminary experiments indicated that WICS induced a transient inflammatory response in control mice, whereas inflammatory cytokine expression persisted in SITH-1 mice during the recovery period.

### 2.5 Drugs

The Melsmon® preparation was kindly provided by Melsmon Pharmaceutical Co., Ltd (Tokyo, Japan). The vehicle for diluting Melsmon was prepared by mixing the following components: 65 mM NaCl, 198 μM CaCl_2_, 129 μM MgCl_2_, 7.09 μM FeCl_2_, and 1.16 mM phosphate buffer (pH 7.0), followed by sterile filtration.

### 2.6 Administration of melsmon preparation

**FIGURE 1:**
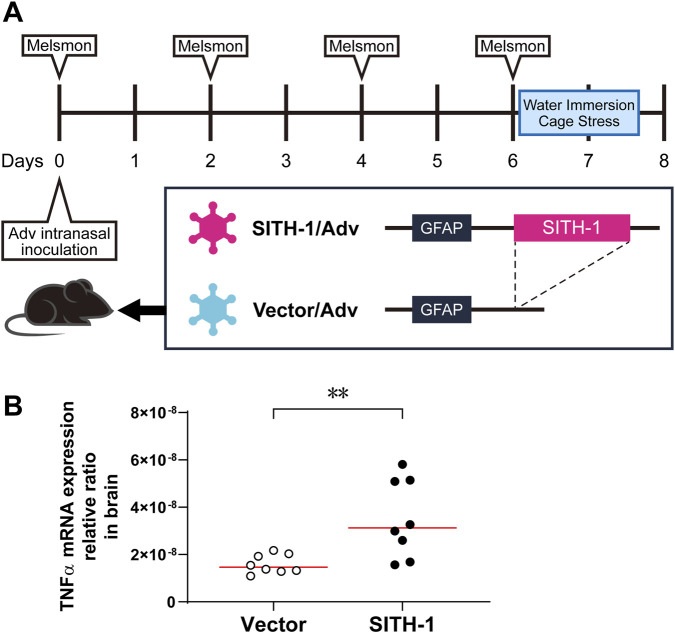
Experimental protocol and elevation of brain inflammatory cytokines in SITH-1 mice following water immersion cage stress (WICS). **(A)** Schematic representation of the experimental protocol used in this study. Recombinant adenovirus vectors were intranasally administered, and WICS was initiated 6 days later. After 18 h of rest in the home cage, tissue samples were collected. For Melsmon treatment experiments, intramuscular injections were administered on days 0, 2, 4, and 6. **(B)** TNFα mRNA expression in whole brain tissue. TNFα expression in the brains of SITH-1 mice (n = 8) was significantly increased after WICS compared to control mice (n = 8). Bars represent median values. Mann–Whitney U test. **p < 0.01. Data represent pooled results from two independent experiments (4 mice per group per experiment).

For Melsmon-treated mice, a total volume of 50 μL containing 20 μL of Melsmon and 30 μL of Melsmon vehicle was injected intramuscularly into the hind limb every other day (Figure 1A). Control mice received 50 μL of the Melsmon vehicle alone via the same route and schedule. The dose of Melsmon administered in this study was determined with reference to previously published *in vivo* studies using Melsmon (Park et al., 2010; Oh, Jung and Sul, 2023), in which 20 μL was administered intramuscularly under comparable experimental conditions.

### 2.7 Real-time PCR

Total RNA was extracted from animal tissues using the RNeasy Mini QIAcube Kit (Qiagen, Hilden, Germany). For brain samples, whole brain tissue was collected and used for RNA extraction. Complementary DNA (cDNA) was synthesized from total RNA using the PrimeScript RT Reagent Kit (Takara Bio Inc., Shiga, Japan). Quantification of mRNA levels was performed using Premix Ex Taq (Perfect Real Time; Takara Bio Inc.) on a QuantStudio Real-Time PCR System (Thermo Fisher Scientific, Waltham, MA, United States). The thermal cycling conditions were as follows: 95 °C for 30 s, followed by 45 cycles of 95 °C for 5 s and 60 °C for 31 s. Gene expression analyses were performed by a technician who was blinded to the experimental conditions. Data analysis was conducted using QuantStudio Design and Analysis Software (Thermo Fisher Scientific). The following TaqMan Gene Expression Assays (Thermo Fisher Scientific) were used: mouse TNF-α (Mm00443258_m1), mouse StAR (Mm00441558_m1), mouse IL-6 (Mm00446190_m1), and eukaryotic 18 S rRNA (Hs99999901_s1) as the internal control.

### 2.8 Statistical analysis

Statistical analyses were performed using GraphPad Prism software (GraphPad Software, San Diego, CA, United States). Statistical outliers were excluded using the software. For comparisons between two groups, the non-parametric Mann–Whitney U test was used. For comparisons among multiple groups, the Kruskal–Wallis test followed by Dunn’s *post hoc* test was employed. Red horizontal bars in the figures represent median values. A p-value of less than 0.05 was considered statistically significant.

## 3 Results

### 3.1 Induction of neuroinflammatory cytokine expression in the brains of SITH-1 mice by water immersion cage stress (WICS)

To investigate whether neuroinflammatory cytokines are induced in SITH-1 mice, which exhibit depression-like behavior under mild stress conditions, we administered an adenovirus encoding SITH-1 under an astrocyte-specific promoter via intranasal inoculation. Control mice were treated with an empty adenoviral vector. Six days after inoculation, mice were subjected to 30 h of water immersion cage stress (WICS), and brain tissues were collected 18 h after the end of stress exposure (Figure 1A). Quantitative reverse transcription real-time PCR (qRT-PCR) analysis revealed that the expression of the proinflammatory cytokine TNF-α was significantly increased in the brains of SITH-1 mice following WICS (Figure 1B). Based on this result, we next investigated whether administration of the human placental extract Melsmon could suppress this stress-induced cytokine expression.

### 3.2 Comparison of neuroinflammatory cytokine and adrenal StAR expression between melsmon-treated and untreated SITH-1 mice

**FIGURE 2:**
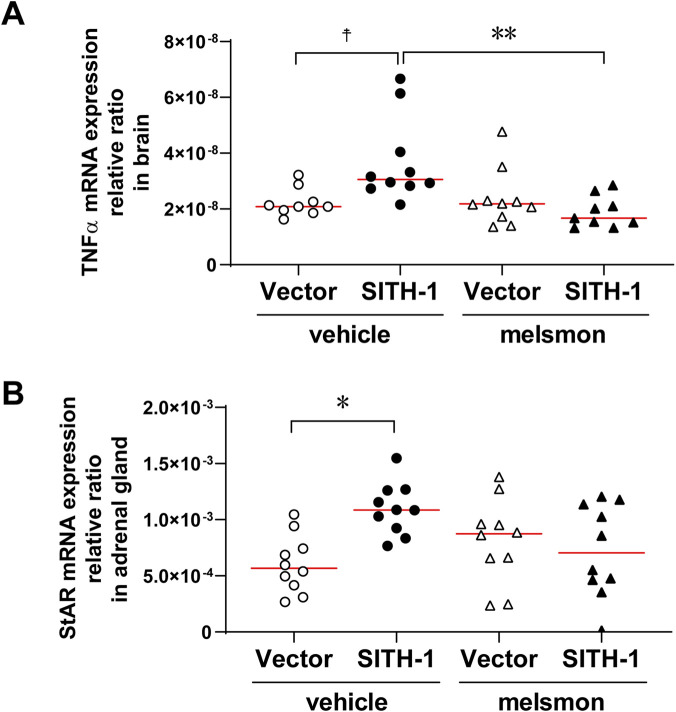
Effects of Melsmon treatment on brain TNFα and adrenal StAR expression in WICS-exposed SITH-1 mice. **(A)** TNFα mRNA expression in whole brain tissue. WICS induced an increase in TNFα expression in the brains of vehicle-treated SITH-1 mice, whereas Melsmon administration significantly suppressed this elevation. Bars represent median values. Kruskal–Wallis test followed by Dunn’s *post hoc* test. ☨p < 0.1; **p < 0.01. Group sizes were as follows: Vector + Vehicle (n = 9), SITH-1 + Vehicle (n = 10), Vector + Melsmon (n = 10), and SITH-1 + Melsmon (n = 9). Data represent pooled results from two independent experiments (4-5 mice per group per experiment). **(B)** StAR mRNA expression in adrenal gland tissue. WICS significantly increased adrenal StAR expression in vehicle-treated SITH-1 mice, but this elevation was not observed in the Melsmon-treated group. Bars represent median values. Kruskal–Wallis test followed by Dunn’s *post hoc* test. *p < 0.05. Group sizes were as follows: Vector + Vehicle (n = 10), SITH-1 + Vehicle (n = 10), Vector + Melsmon (n = 10), and SITH-1 + Melsmon (n = 10). Data represent pooled results from two independent experiments (5 mice per group per experiment).

To evaluate the effect of Melsmon on stress-induced neuroinflammation, SITH-1 mice were intramuscularly administered Melsmon every other day for a total of four doses prior to WICS exposure (Figure 1A). Following WICS, brain tissues were analyzed, and the elevated TNF-α expression observed in the brains of SITH-1 mice was significantly reduced in the Melsmon-treated group (Figure 2A). In addition, expression of the steroidogenic acute regulatory protein (StAR), a marker of HPA axis activation in the adrenal glands, was also decreased by Melsmon administration (Figure 2B).

### 3.3 Comparison of peripheral inflammatory cytokine expression between melsmon-treated and untreated SITH-1 mice

**FIGURE 3:**
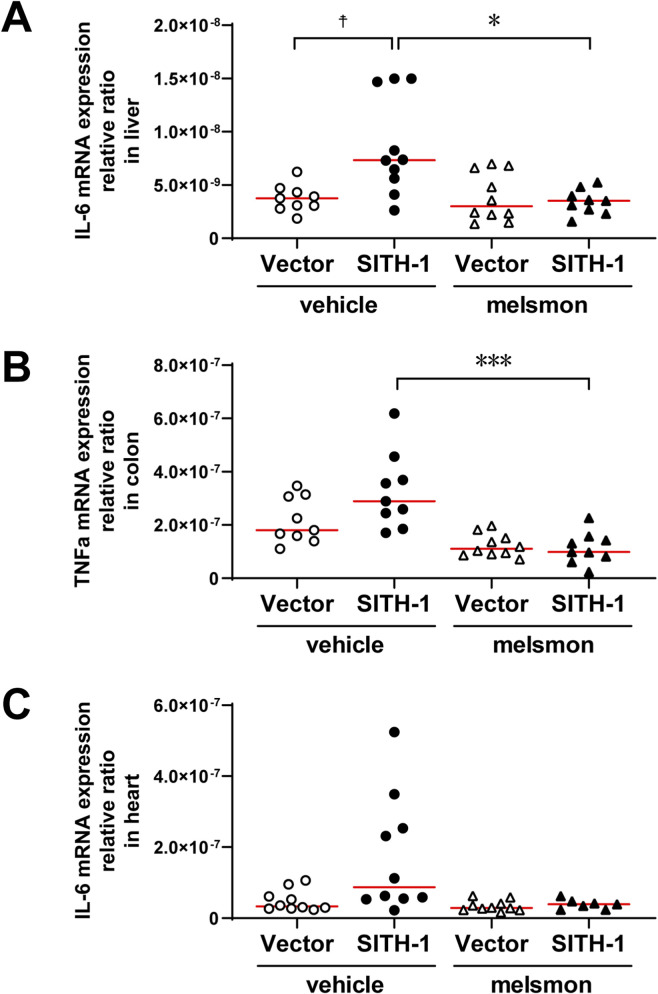
Effects of Melsmon treatment on inflammatory cytokine expression in the liver, colon, and heart of WICS-exposed SITH-1 mice. **(A)** IL-6 expression in the liver of vehicle-treated SITH-1 mice showed an increasing trend after WICS, which was significantly suppressed by Melsmon treatment. Bars represent median values. Kruskal–Wallis test followed by Dunn’s *post hoc* test. ☨p < 0.1; *p < 0.05. Group sizes were as follows: Vector + Vehicle (n = 9), SITH-1 + Vehicle (n = 10), Vector + Melsmon (n = 10), and SITH-1 + Melsmon (n = 9). Data represent pooled results from two independent experiments (4-5 mice per group per experiment). **(B)** TNFα expression in the colon was increased in vehicle-treated SITH-1 mice following WICS and was significantly reduced by Melsmon treatment. Bars represent median values. Kruskal–Wallis test followed by Dunn’s *post hoc* test. ***p < 0.001. Group sizes were as follows: Vector + Vehicle (n = 9), SITH-1 + Vehicle (n = 9), Vector + Melsmon (n = 10), and SITH-1 + Melsmon (n = 9). Data represent pooled results from two independent experiments (4-5 mice per group per experiment). **(C)** IL-6 expression in the heart was elevated after WICS in vehicle-treated SITH-1 mice, and this was attenuated by Melsmon treatment. Bars represent median values. Kruskal–Wallis test followed by Dunn’s *post hoc* test. Group sizes were as follows: Vector + Vehicle (n = 10), SITH-1 + Vehicle (n = 10), Vector + Melsmon (n = 10), and SITH-1 + Melsmon (n = 7). Data represent pooled results from two independent experiments (3-5 mice per group per experiment).

Chronic activation of the HPA axis observed in SITH-1 mice has been reported to promote inflammatory cytokine production in peripheral organs (Zunszain et al., 2011; Walsh, Bovbjerg and Marsland, 2021). Moreover, peripheral inflammation can in turn promote neuroinflammation (Kempuraj et al., 2017; Hassamal, 2023). Therefore, we examined whether Melsmon could attenuate inflammatory cytokine expression in peripheral organs of WICS-exposed SITH-1 mice. qRT-PCR analysis revealed that WICS-induced expression of IL-6 in the liver (Figure 3A), TNF-α in the colon (Figure 3B), and IL-6 in the heart (Figure 3C) were all reduced in the Melsmon-treated group.

## 4 Discussion

In the present study, we demonstrated that 18 h after exposure to water immersion cage stress (WICS), inflammatory cytokine expression was elevated in both the brain (Figures 1, 2) and peripheral organs (Figure 3) of a stress-sensitive depression model mouse expressing SITH-1 specifically in astrocytes. To our knowledge, this is the first report showing that, in this stress-sensitive model, inflammatory responses are concurrently induced and maintained in both central and peripheral tissues following stress exposure. These findings support the concept that neuroinflammation and systemic inflammation may be bidirectionally involved in the pathophysiology of depression. In the present experimental design, tissues were collected 18 h after WICS exposure to allow transient stress responses to subside. Under these conditions, inflammatory responses remained elevated in SITH-1 mice, whereas they were attenuated in control mice, suggesting sustained inflammatory activation in the stress-vulnerable state.

Administration of the human placental extract Melsmon significantly suppressed not only neuroinflammatory cytokine expression in the brain but also the expression of steroidogenic acute regulatory protein (StAR) in the adrenal gland. StAR is a key rate-limiting factor in adrenal steroidogenesis, and its reduced expression suggests attenuation of HPA-axis hyperactivation. Inflammatory cytokine receptors are expressed in HPA-axis–related structures such as the hypothalamus and pituitary gland, and centrally produced inflammatory cytokines have been shown to stimulate corticotropin-releasing hormone (CRH) neurons and pituitary corticotrophs, thereby promoting the secretion of adrenocorticotropic hormone (ACTH) and glucocorticoids (Turnbull and Rivier, 1995; Gądek-Michalska et al., 2013). Thus, the HPA-axis hyperactivation observed in SITH-1 mice may be driven, at least in part, by neuroinflammatory cytokines. Our findings suggest that Melsmon may contribute to the maintenance of HPA-axis homeostasis through suppression of stress-induced neuroinflammation.

Notably, Melsmon administration also reduced inflammatory cytokine expression in peripheral organs, including the liver, colon, and heart (Figure 3). Chronic stress is known to induce inflammatory cytokine production in these organs (Mann, 2003; Miller et al., 2019; Wei et al., 2019). However, in SITH-1 mice, elevated inflammatory cytokine expression persisted in peripheral tissues even after a single stress exposure, and this sustained inflammatory response was suppressed by Melsmon treatment. Notably, this sustained inflammatory response was observed after a single episode of stress exposure, suggesting that SITH-1 expression may prolong stress-induced inflammatory activation beyond the transient responses typically observed in control animals. Peripheral inflammation has been reported to induce neuroinflammatory cytokine expression through humoral and neural pathways (Raghavendra et al., 2004; Silverman et al., 2015; Talley et al., 2021). Taken together, our results suggest that Melsmon may interrupt the vicious cycle linking stress, neuroinflammation, and peripheral inflammation.

Various biological functions of human placental extracts have been reported, including anti-inflammatory activity (Alimu, Yamamoto, and Nakahata, 2025). For example, *in vitro* studies have shown that one placental extract, Laennec, induces macrophage polarization toward an anti-inflammatory M2 phenotype (Ishikawa et al., 2025). In addition, both Laennec and Melsmon have been reported to exert anti-inflammatory effects through antioxidant mechanisms. Laennec treatment increases the expression of antioxidant genes such as *Hmox1*, *Nqo1*, *Cat*, and *Sod1*, and enhances the activity of nuclear factor erythroid 2–related factor 2 (NRF2), a key regulator of antioxidant pathways (Yamauchi et al., 2020). Similarly, Melsmon has been shown to upregulate antioxidant genes including *CYGB*, *APOE*, *NQO1*, and *PTGS1*, and to increase NRF2 protein levels (Huang et al., 2022). NRF2 activation has been reported to suppress the expression of inflammatory cytokines such as IL-6 and IL-1β (Kobayashi et al., 2016), which may counteract oxidative stress–induced activation of transcription factors such as NF-κB and AP-1 that promote inflammation (Chatterjee, 2016; Liu et al., 2023). Although the present study did not directly assess antioxidant gene expression or NRF2 activity, the observed suppression of inflammatory cytokines in both central and peripheral tissues, along with reduced adrenal StAR expression, is consistent with the involvement of antioxidant pathways.

Clinical studies in humans have reported that continuous administration of Melsmon alleviates depressive mood, anxiety, and fatigue-related symptoms (Kovalenko and Atalyan, 2016). The anti-inflammatory effects observed in the present study are therefore consistent with these reported clinical benefits.

Several limitations of this study should be acknowledged. First, inflammatory cytokine expression was assessed only at the mRNA level, and protein expression and cellular localization were not examined. Second, HPA-axis activation was evaluated based on adrenal StAR mRNA expression as a molecular marker of steroidogenic activity. Circulating corticosterone levels were not measured, and the chronicity of HPA-axis activation was not directly assessed. Future studies incorporating endocrine measurements will be necessary to further characterize HPA-axis dynamics in this model. Third, the present analysis focused primarily on molecular endpoints, and functional outcomes such as changes in neuroplasticity markers or depression-like behaviors were not evaluated. Fourth, although no obvious intestinal bleeding or severe physical abnormalities were observed during the experiment, potential stress-related pathological changes such as gastric ulcer formation were not systematically evaluated and therefore cannot be excluded. Future studies incorporating behavioral assessments, analyses of neuronal structural plasticity, and comprehensive investigation of molecular mechanisms, including antioxidant pathways, will be required to further clarify the therapeutic potential of Melsmon in psychiatric disorders. In addition, although both Melsmon and Laennec have been reported to enhance NRF2 activity and suppress inflammatory cytokine expression, the specific bioactive components responsible for NRF2 activation remain unidentified. Despite differences in manufacturing processes, both preparations involve acid hydrolysis of human placenta (Alimu, Yamamoto, and Nakahata, 2025), suggesting that acid-hydrolyzed placental components may contribute to NRF2 activation. Elucidating these active constituents will be an important goal for future research.

In conclusion, this study demonstrates that Melsmon exerts multifaceted suppressive effects on stress-induced neuroinflammation and peripheral inflammation, thereby highlighting a novel therapeutic potential of human placental extracts for stress-related psychiatric disorders.

## Data Availability

The original contributions presented in this study are included in the article. Further inquiries can be directed to the corresponding author.
